# Gender Equality in Antiphospholipid Syndrome Publications: A Comprehensive Analysis of First Author Trends

**DOI:** 10.7759/cureus.50186

**Published:** 2023-12-08

**Authors:** Mahima Kuruvila, Eden Estevez, Aruna Anantharaj, Anjali Mediboina

**Affiliations:** 1 Internal Medicine, Caribbean Medical University School of Medicine, Chicago, USA; 2 Internal Medicine, St. George’s University School of Medicine, St. George’s, GRD; 3 Internal Medicine, Wuhan University School of Medicine, Wuhan, CHN; 4 Community Medicine, Alluri Sitarama Raju Academy of Medical Sciences, Eluru, IND

**Keywords:** bibliometric analysis, pubmed, gender trends, antiphospholipid antibody syndrome, antiphospholipid antibody, aps, apla

## Abstract

Antiphospholipid syndrome (APS) is a condition characterized by the production of procoagulant antibodies, which in turn increases the risk of thrombotic events in multiple blood vessels and is associated with recurrent miscarriages and premature births. The study aimed to identify and analyze the gender trends of the first authors in PubMed-indexed publications related to APS. The present cross-sectional study analyzed all PubMed-indexed articles published between January 2018 and December 2022. All articles with the term "antiphospholipid syndrome" in their titles were included in the study. Full names and countries were used to determine the gender of the author in the NamSor application program interface (API) and statistical analysis was done using R software version 4.3.1 (R Core Team, Vienna, Austria) and the Autoregressive Integrated Moving Average (ARIMA) model. Finally, a total of 1176 articles were evaluated in the study after the inclusion and exclusion criteria were applied. The highest number of publications by female first authors was in the year 2022 with a total of 132 articles published. Forecasting predicts that by 2027, approximately 122 articles will be published by male authors and 132 articles will be published by female authors. The highest female-to-male publication ratio is seen in Russia, with a ratio of 2, followed closely by Poland (1.86) and Greece (1.85). In conclusion, gender equality has not significantly improved in the field of APS research. Thus, the gender gaps must be addressed, to advance the medical field, improve patient care, and ultimately contribute to better health outcomes for women worldwide.

## Introduction and background

Antiphospholipid syndrome (APS) is a complex autoimmune condition characterized by the production of procoagulant antibodies, leading to an increased risk of thrombotic events in multiple blood vessels [1]. APS is associated with recurrent vascular occlusion and organ damage, with an estimated prevalence of 50 in 100,000 people in the general population, and is also closely linked to pregnancy morbidity, such as recurrent miscarriages and premature births [1]. APS can occur idiopathically or secondary to autoimmune diseases, such as systemic lupus erythematosus (SLE) [2].

The complexities of APS extend beyond its clinical manifestations and prevalence rates; gender dynamics also play a critical role in exploring the intricacies of this condition. Recent papers by Truglia et al., Moschetti et al., and de Carvalho have found a female preponderance in terms of epidemiology and have also found gender to influence clinical outcomes; this is postulated to be due to the influence of sex hormones and the occurrence of pregnancy [3-5]. Gender disparities can affect disease presentation, symptomatology, and prognosis of any disease, and studies such as those by Alcalde-Rubio et al. have consistently highlighted the delayed diagnoses experienced by women across various health conditions [6]. This becomes particularly evident in conditions like APS, where the impact can be compounded due to specific complications related to pregnancy, recurrent miscarriages, and premature births [3].

Understanding gender disparities in healthcare and their impact on disease management for women emphasizes the critical need for gender-inclusive perspectives in research. Investigating the gender representation among authors involved in APS research becomes crucial as it reflects not only the scientific landscape but also the inclusivity of perspectives influencing disease understanding and management. A deeper exploration of gender trends in authorship can shed light on potential biases or disparities that might affect research directions, knowledge dissemination, and ultimately, patient care [7].

Moreover, gender disparities in publishing, particularly the lack of parity between author genders, lead to imbalances across various academic domains. The relationship between publication and career advancement often extends to disparities in grants, scholarships, laboratory development, publication support, and clinical exposure. As evident through the volume of publications and the author order, notably the senior author position, gender imbalances significantly impact various academic realms [8]. Therefore, examining the gender trends in APS publications is poised to provide valuable insights into broader aspects of autoimmune research and women's representation within this field. Our study aimed to conduct a concise review of gender trends in medical research and discern differences among published author rankings, aiming to address the broader implications of gender disparities in academia.

Thus, this study aimed to conduct a comprehensive analysis of gender trends in first authors of publications related to APS, as found in the PubMed database. The gender trends of the first author rank of these publications reflect the education and training of women and would be helpful to identify patterns for gender differences by allowing us to understand how this contributes to variations in female presence in science. 

The overall aims of this study were to examine the gender disparity among first authors in publications related to antiphospholipid syndrome (APS) from the PubMed-indexed database; to further analyze the patterns and associations in publications, consider variables including the country of the first author, year of publication, and journal of publication; and to utilize predictive statistical methods to forecast future trends in gender distribution among the first authors in the field of antiphospholipid syndrome research.

## Review

Methods

Design and Setting

This retrospective bibliometric study was designed to explore gender trends in the first authors in publications related to antiphospholipid syndrome (APS). The study was conducted on May 9, 2023, and involved a systematic analysis of PubMed-indexed publications over five years, from January 1, 2018, to December 31, 2022.

The PubMed database was selected as the primary data source due to its extensive collection of multidisciplinary peer-reviewed published literature, and its free accessibility, ensuring open access to the widest audience, unlike some other databases that may have restricted access or limited coverage. Data collection was done via a systematic search using the term “antiphospholipid syndrome.” The publication date range was specified from January 1, 2018, to December 31, 2022, to focus on recent literature. The search results were sorted from most recent to least recent, and the entire list of articles was downloaded as a .csv file. Subsequently, the full names of the first authors and their respective countries of origin were collected, with country determination based on their institutional affiliations.

All publications that had "antiphospholipid syndrome" in their titles were included in the study, while those articles published outside of the selected time frame (before 2018 and after 2022) and articles without the full names of the first authors were excluded (Table 1).

**Table 1:** Inclusion and exclusion criteria applied.

Inclusion criteria	Exclusion criteria
All articles with “antiphospholipid syndrome” in the title	Articles published outside of the selected time frame (before 2018 and after 2022)
All research papers investigating APS and published between 2018 and 2022	Articles without the full names of the first authors

Gender Determination and Country Affiliations

Determination of the gender of the first authors and their respective country affiliations was done via the NamSor application program interface (API), which provides a gender identification based on an individual's full name and country of origin [9]. The authors' country affiliations were determined based on their institutional affiliations. Previous studies, such as those by Tricco et al. and Morgan et al., have demonstrated the reliability of the NamSor API for determining the gender of authors [10,11]. NamSor provides a probability range (0 to 1) of an individual’s gender. Those genders with a probability of 0.6 and below were manually cross-checked with the author’s professional profiles (Research Gate, LinkedIn, Institutional profiles), and corrected by the researchers. Those genders that remained unidentified were ultimately discarded.

Data Entry and Statistical Analysis

The retrieved data was exported to Microsoft Excel, where it was structured into multiple columns representing the various elements of the articles including titles, publication years, first author names, full names of the first authors, gender of the first authors, affiliations of the first authors (i.e., by region/country), journal names, and publication year.

R software version 4.3.1 (R Core Team, Vienna, Austria), which is a widely utilized tool for statistical analysis and data visualization, was employed to analyze gender trends, forecast future patterns within the dataset, and make informed predictions about future patterns. An autoregressive integrated moving average (ARIMA) model was utilized for time series analysis. This enabled the identification of underlying patterns and trends in the data, facilitating predictions of the number of articles likely to be authored by male and female first authors up to 2027. It is noteworthy that although the data for 2023 were incomplete at the time of the study, they were thoughtfully included in the prediction model to provide a more immediate forecast, with a clear acknowledgment of the limitations arising from incomplete data.

Additionally, a Fisher's exact test was performed to explore the association between the variables of gender and country. This statistical test was chosen for its suitability in analyzing tables with small sample sizes and provides exact p-values, regardless of the sample size or data distribution. To enhance the presentation of the results, graphical representations were created using Datawrapper, ensuring a comprehensive and visually accessible depiction of the findings.

Results

A total of 1200 articles were collected for data analysis, of which 24 were excluded due to the lack of full names in these articles. Thus, a total of 1176 articles were analyzed in our study. Out of these, the first authors included 558 (47.4%) females and 618 (52.6%) males.

**Figure 1:**
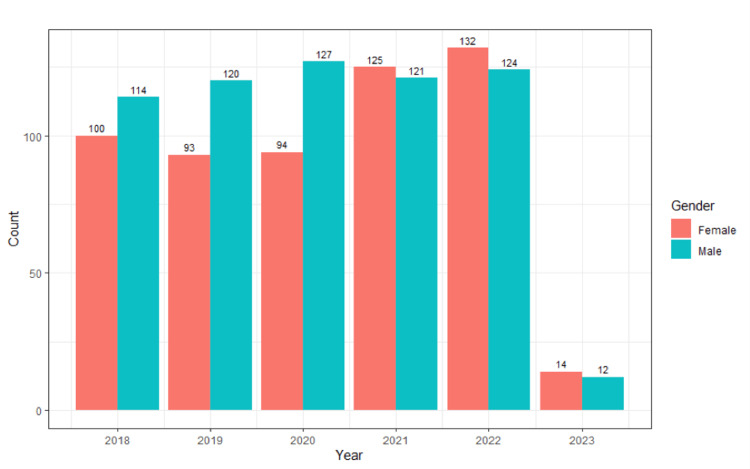
The total number of male and female authors each year, ranging from 2018 to 2023.

Figure 1 shows the total number of male and female authors each year, ranging from 2018 to 2023. The highest number of publications by female first authors was in the year 2022 with a total of 132 articles published.

**Figure 2:**
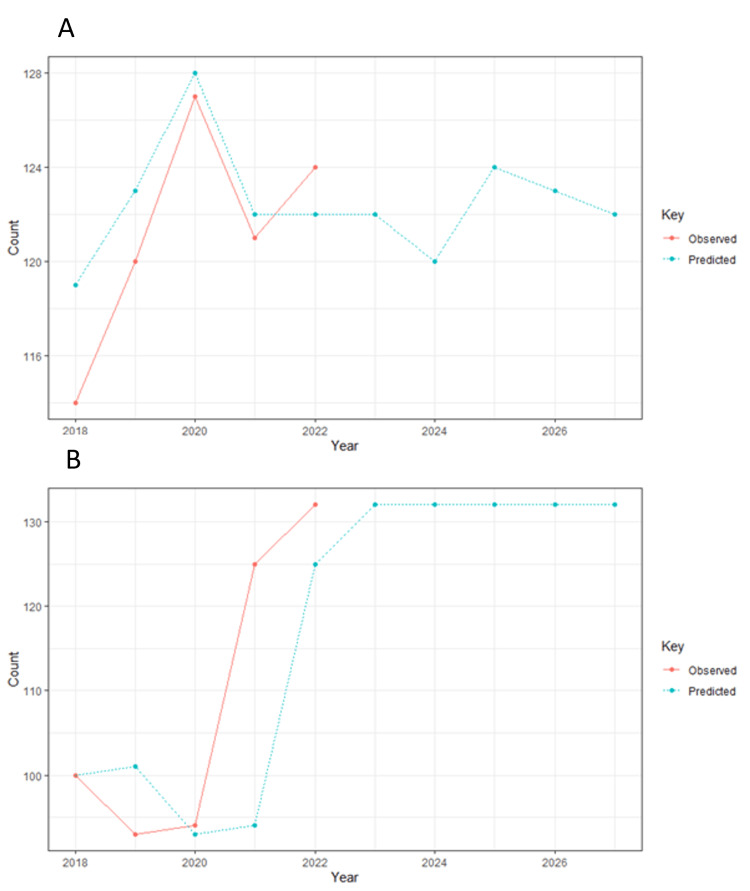
The prediction of publication trends of male and female authors for the next five years, with modeling data taken from 2018 to 2022 (A) and forecasting done from 2018 to 2027 (B).

Figures 2A, 2B show the prediction of publication trends of male and female authors for the next five years, with modeling data taken from 2018 to 2022 and forecasting done from 2018 to 2027. It is expected that in the year 2027, approximately 122 articles will be published by male authors and 132 articles will be published by female authors.

Table 2 shows the gender trends in publications based on various countries. The highest female-to-male publication ratio is seen in Russia, with a ratio of 2, followed closely by Poland (1.86) and Greece (1.85).

**Table 2:** Top 10 journals having favorable gender ratio (with more than 10 publications in total).

Journal/book	Female	Male	Ratio
Journal of Thrombosis and Thrombolysis	8	5	1.6
Clinical and Experimental Rheumatology	9	7	1.29
Thrombosis Research	19	15	1.27
Clinical Rheumatology	12	10	1.2
Journal of Clinical Medicine	6	5	1.2
Frontiers in Immunology	20	18	1.11
Lupus	46	47	0.98
Journal of Thrombosis and Haemostasis	14	16	0.88
Seminars in Thrombosis and Hemostasis	8	10	0.8
Journal of Autoimmunity	6	8	0.75

Table 3 shows the top 10 journals having favorable gender ratios with more than 10 publications in total. Journal of Thrombosis and Thrombolysis has the most favorable ratio (1.6), with 23 publications by female first authors, followed by Clinical and Experimental Rheumatology (1.29), with nine publications by female first authors.

**Table 3:** Top 10 journals having high gender ratio (with more than 10 publications in total).

Journal/book	Female	Male	Ratio
International Journal of Molecular Sciences	9	3	3
Autoimmunity Reviews	20	9	2.22
Current Rheumatology Reports	8	4	2
Journal of Thrombosis and Thrombolysis	8	5	1.6
Clinical and Experimental Rheumatology	9	7	1.29
Thrombosis Research	19	15	1.27
Clinical Rheumatology	12	10	1.2
Journal of Clinical Medicine	6	5	1.2
Frontiers in Immunology	20	18	1.11
Lupus	46	47	0.98

Table 4 shows the top 10 journals with high gender ratios, with more than 10 publications in total. The International Journal of Molecular Sciences has the highest ratio (3) with eight publications by female first authors, followed by Autoimmunity Reviews (2.22) with 13 publications by female first authors.

**Table 4:** Top 10 countries having high gender ratio (with more than 10 publications in total).

Country of first author	Female	Male	Ratio
Russia	10	5	2
Poland	13	7	1.86
Greece	24	13	1.85
Canada	11	7	1.57
China	67	48	1.4
Italy	63	53	1.19
Turkey	11	11	1
Belgium	6	6	1
South Korea	9	10	0.9
Netherlands	16	18	0.89

The statistical analysis was conducted using Fisher's exact test to examine the association between gender and country variables. The results revealed a highly significant association (p < 0.001) between gender and country, indicating a strong relationship between these two factors.

**Figure 3:**
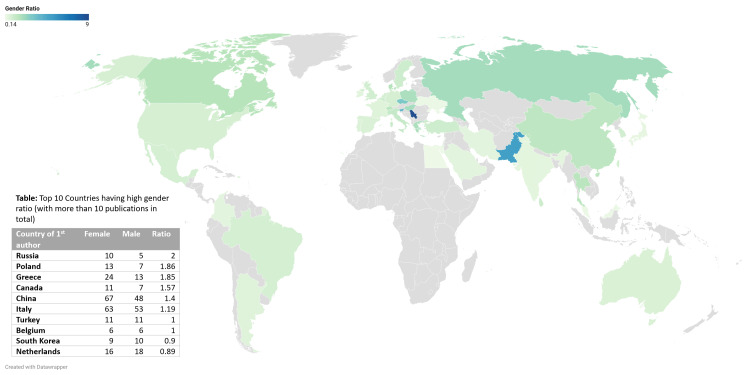
The top 10 countries with the highest gender ratios, with more than 10 publications in total.

Figure 3 also shows the top 10 countries with the highest gender ratios, with more than 10 publications in total. Russia has the highest ratio (2) of female first authors, followed by Poland (1.86) and Greece (1.85).

Table 5 shows the top 10 female authors with the highest number of publications. Tektonidou published the highest number of publications with nine articles in this study from Greece, followed by Hannah Cohen in the United Kingdom and Amelia Ruffatti in Italy with seven publications each.

**Table 5:** The top 10 female authors with the highest number of publications.

First author	Full name of first author	Institution	Country of first author		Publications (n)
Tektonidou MG	Maria G Tektonidou	University of Athens Medical School	Greece	9	
Cohen H	Hannah Cohen	University College London	UK	7	
Ruffatti A	Amelia Ruffatti	University of Padua	Italy	7	
Yelnik CM	Cecile M Yelnik	National Reference Center for Rare Systemic and Autoimmune Diseases, European Reference Network on Rare Connective Tissue and Musculoskeletal Diseases (ReCONNECT)	France	5	
Medina G	Gabriela Medina	La Raza National Medical Center	Mexico	4	
Djokovic A	Aleksandra Djokovic	University of Belgrade	Serbia	4	
Schreiber K	Karen Schreiber	Institute for Regional Health Research, University of Southern Denmark	Denmark	4	
Erton ZB	Zeynep B Erton	Hospital for Special Surgery, New York	USA	3	
Sayar Z	Zara Sayar	University College London Hospitals NHS Foundation Trust, London	UK	3	
Xourgia E	Eleni Xourgia	National and Kapodistrian University of Athens	Greece	3	

Discussion

This study aimed to investigate and analyze the gender trends of first authors in publications related to Antiphospholipid syndrome from PubMed-indexed articles spanning from January 1, 2018, to December 31, 2022. The study utilized the Namsor API to determine the gender of authors based on various factors such as country of origin, ethnicity, and names. The findings of this study shed light on the gender distribution of first authors in this particular field and provide insights into gender trends over time, across countries, and within specific journals.

While the number of women in medicine and medical research has been increasing throughout the years, studies such as Baobeid et al. and Bernardi et al., have reported a higher number of male authors occupying the first and last authorship positions in medical research papers [12,13]. Furthermore, within specialized medical areas like hand surgery, spine surgery, and otolaryngology, there has been an encouraging increase in the number of women engaging in research. However, it is essential to note that despite this progress, there is still a significant gender gap in authorship in these fields [14-16].

The present study suggests a relatively balanced gender distribution in terms of first authors within the scope of the study, with 47.4% of the papers authored by females and 52.6% authored by males; hence suggesting that, despite the disparities, there is progress in gender representation.

Examining gender distribution across countries, this study revealed that Russia had the highest female-to-male publication ratio, followed closely by Poland and Greece. The statistical analysis, using Fisher's exact test, also confirmed a significant association between gender and country, indicating variations in gender representation across nations. Goryunova et al. found that Russia and Eastern European nations exhibited a greater presence of female senior leaders, and this might indicate a societal acceptance of women in influential roles, fostering gender parity in academic and research settings within these regions [17]. These findings therefore underscore the pivotal role of socio-cultural factors in shaping gender equity within scientific research. They also suggest that nations proactively addressing gender disparities and implementing supportive policies for women's advancement are more likely to exhibit higher proportions of female authors in research [18,19].

The present study also highlights a notable male-to-female ratio in various journals, which may signal underlying systemic issues in academic publishing. While journals with stringent acceptance rates and blinded peer review processes aim for rigorous standards and reduced gender bias, they may inadvertently hinder certain groups, including women, from publication. These disparities underscore the need to address gender imbalances and enhance opportunities for female researchers in APS-related studies to ensure equitable contributions to APS knowledge [20].

The table featuring the top 10 female authors with the highest publication counts was included and serves to recognize and celebrate the significant contributions of women in the field of APS-related literature, with Tektonidou from Greece having nine publications.

The analysis of gender trends over time demonstrated a notable increase in the number of publications by female first authors in the year 2022, which indicates a positive shift towards greater representation and involvement of female researchers regarding antiphospholipid syndrome. Furthermore, the forecasting model predicts a continued rise in the number of publications by both male and female authors in the coming years, aligning with observations made by Bernardi et al., who determined a narrowed and further reduced gender gap in publication trends [13].

Limitations

While the study provides valuable insights into the gender trends of first authors in publications related to antiphospholipid syndrome, it is important to recognize several limitations when interpreting the findings of the study and consider them as avenues for future research to further enhance our understanding of gender representation in medical research.

Firstly, the study primarily focuses on the quantitative analysis of gender representation and trends, not delving into the underlying reasons or factors contributing to the observed patterns. Thus, a qualitative analysis could provide deeper insights into the barriers, challenges, and experiences faced by female authors in the field of antiphospholipid syndrome. Additionally, it is important to note that the study uses past trends for prediction, which may not accurately reflect future changes. Future research employing more dynamic models or accounting for evolving sociocultural factors could offer more robust predictions regarding gender representation in APS-related publications. 

Secondly, only those publications related to antiphospholipid syndrome from PubMed-indexed articles within a specific time frame were included. This narrow scope may not fully capture the broader gender distribution and trends in medical research as a whole. Furthermore, it is possible that certain publications, particularly those from non-indexed or non-English language sources, are not captured in the analysis, which could influence the overall gender distribution observed.

Lastly, while the NamSor API has been previously used in various studies to determine the gender of authors based on different factors, it must be acknowledged that the accuracy of gender determination algorithms can vary, and there may be instances of misclassification or limitations in capturing gender identities accurately. It also must be acknowledged that such an approach may inadvertently exclude gender non-binary and transgender scientists. This exclusion overlooks the diverse spectrum of gender identities within the scientific community. It is essential to acknowledge and address the complexities of gender diversity is essential not only to ensure accurate representation but also to prevent potential gender discrimination and maintain accountability among leaders in the field.

Potential Strategies

Despite these limitations, our study underscores the importance of achieving gender parity in publishing, which, in turn, can contribute to more equitable academic landscapes and research environments. To address the persisting gender disparities in academic publishing, a multifaceted approach is crucial. Firstly, institutions and journals should proactively implement policies aimed at fostering equal representation on editorial boards and review panels, ensuring that a diverse range of perspectives is taken into account. Auelua-Toomey and Roberts have noted that the lack of racial diversity among editors at mainstream journals can hinder the advancement of scholarship in various domains, signaling to scholars that their research may not be valued [21]. Thus, inclusive editorial practices are essential for closing gender and racial gaps in academic publishing. 

It is also important to ensure a more accurate representation of all gender identities in scientific contributions, which could involve providing authors the option to voluntarily disclose their personal pronouns and gender identities during the submission process. Journals could consider incorporating fields for gender identity alongside other author information to create a more comprehensive census. By actively inviting authors to share their gender identities, publications can enhance inclusivity and contribute to fostering an environment of respect and equity within academic publishing.

Secondly, mentorship programs and initiatives designed to support female researchers in their careers can play a pivotal role in narrowing the gender gap. As supported by Wasburn's research, collaborative, peer-oriented mentoring models have proven particularly effective for women seeking further career advancement [22]. These programs provide invaluable guidance and opportunities for skill development, ultimately contributing to increased representation of women in leadership positions within academia.

Lastly, creating an inclusive research environment where researchers of all genders have equitable access to funding, resources, and opportunities is paramount. Steinþórsdóttir et al. have emphasized that competitive grants often exhibit bias, favoring not only men but also male-dominated fields [23]. Rectifying these biases ensures a level playing field, allowing researchers of all genders to thrive and contribute meaningfully to academic publishing. These strategies can thus collectively work towards addressing gender disparities and fostering a more equitable academic landscape.

## Conclusions

This study concludes that gender equality has not significantly increased. There is a need for a more inclusive environment for female authors. There should be more funding, resources, and facilities available to female authors. This will motivate and encourage them to be involved in publishing as well as being involved at the forefront of academics as well as clinically. Lack of parity between author genders in publishing leads to disparities in other areas of academia, given the relationship between publication and career advancement. These areas may include the distribution of grants and scholarships for conducting research, laboratory development, publication support and backing, and clinical exposure. Moving forward, there is an opportunity for future research endeavors to explore and develop more comprehensive methodologies that embrace greater inclusivity in identifying gender trends within academic literature.

Finally, our study highlights the pressing need for gender equality in publishing and reiterates the need for more specialty-specific analysis regarding gender trends and equality. To achieve this, it is imperative to foster an inclusive environment, provide dedicated resources and funding, and implement supportive policies that empower and encourage female authors. By promoting gender equity in research, we can advance the medical field, improve patient care, and ultimately contribute to better health outcomes for women worldwide.
